# Focal myocardial effects in infective endocarditis

**DOI:** 10.1007/s12471-022-01722-7

**Published:** 2022-09-08

**Authors:** V. A. W. M. Umans, Tj. Germans, M. G. J. Duffels

**Affiliations:** Department of Cardiology, Noordwest Ziekenhuisgroep, Alkmaar, The Netherlands

## Answer

**Fig. 1:**
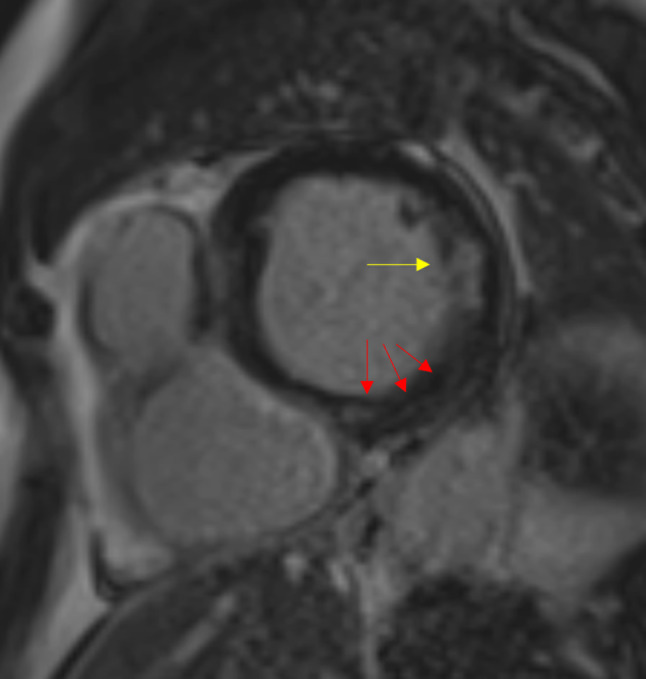
Cardiac magnetic resonance image showing subendocardial lateral myocardial infarction (*yellow arrow*) and foci of myocardial inflammation (*red arrows*)

Given the previously used imaging techniques, we decided to schedule cardiac magnetic resonance imaging (CMR), which showed subendocardial lateral myocardial infarction and foci of myocardial inflammation (Fig. 1). Based on this result, we made the diagnosis of coronary embolisation from endocarditis. This case details two types of myocardial involvement: myocardial ischaemia (embolisation) and interstitial myocarditis (small-vessel vasculitis).

Coronary embolism is a rare complication of infective endocarditis, most prominently originating from large vegetations and infected aortic valves. Large coronary emboli may present as an acute coronary syndrome and are typically seen on coronary angiography as occlusions of the involved coronary artery. Although coronary arteries seem to be protected from embolic involvement, an endocarditis source should be considered [1, 2]. CMR is a sensitive technique to detect cardiac emboli that do not provoke an acute cardiac syndrome [2, 3].

Interstitial myocarditis may originate from seeding of microscopic septic emboli or small-vessel vasculitis [1–3]. The latter may mimic bacterial endocarditis or be an epiphenomenon. Its presence, however, may have profound therapeutic implications. In case of anti-neutrophilic cytoplasmic autoantibodies–mediated vasculitis, switching to steroid treatment should be considered.

This case shows the importance of multimodality imaging in infective endocarditis with elevated troponin levels. Troponin assessment with subsequent CMR may be pivotal to ascertain coronary embolism as the cause of myocardial involvement. Coronary embolism can result from thrombotic or septal embolism. The macroscopic image of myocarditis resulting from septic seeding or, more rarely, from small-vessel vasculitis is provided by CMR, which is currently the method of choice [2, 3].
